# Antiarrhythmic Drug Use in Pregnancy: Considerations and Safety Profiles

**DOI:** 10.3390/jcdd11080243

**Published:** 2024-08-07

**Authors:** Marco Valerio Mariani, Nicola Pierucci, Vincenzo Mirco La Fazia, Pietro Cipollone, Marco Micillo, Andrea D’Amato, Francesca Fanisio, Giuseppe Ammirati, Nethuja Salagundla, Carlo Lavalle, Marco Alfonso Perrone

**Affiliations:** 1 Department of Clinical Internal Anesthesiologic and Cardiovascular Sciences, Sapienza University of Rome, 00161 Rome, Italy; npierucci@gmail.com (N.P.); pietro.cipollone@uniroma1.it (P.C.); andrea.damato@uniroma1.it (A.D.); carlolavalle@yahoo.it (C.L.); 2 Texas Cardiac Arrhythmia Institute, St. David’s Medical Center, Austin, TX 78705, USA; vmirco.lafazia@gmail.com; 3 Cardiology Department, S. Anna University Hospital, 44122 Ferrara, Italy; marco.micillo942@gmail.com; 4 Division of Cardiology, Policlinico Casilino, 00169 Rome, Italy; fanisio.francesca@gmail.com; 5 Department of Advanced Biomedical Sciences, University of Naples Federico II, 80138 Naples, Italy; giuseppe.ammirati92@gmail.com; 6 Texas Health Arlington Memorial Hospital, Dallas, TX 76012, USA; nethuja@gmail.com; 7 Division of Cardiology and CardioLab, Department of Clinical Sciences and Translational Medicine, University of Rome Tor Vergata, 00133 Rome, Italy; marco.perrone@uniroma2.it; 8 Clinical Pathways and Epidemiology Unit, Bambino Gesù Children’s Hospital IRCCS, 00165 Rome, Italy

**Keywords:** antiarrhythmic drugs, arrhythmia, pregnancy

## Abstract

Pregnancy entails notable physiological alterations and hormonal fluctuations that affect the well-being of both the fetus and the mother. Cardiovascular events and arrhythmias are a major concern during pregnancy, especially in women with comorbidities or a history of arrhythmias. This paper provides an overview of the prevalence, therapies, and prognoses of different types of arrhythmias during pregnancy. The administration of antiarrhythmic drugs (AADs) during pregnancy demands careful consideration because of their possible effect on the mother and fetus. AADs can cross the placenta or be present in breast milk, potentially leading to adverse effects such as teratogenicity, growth restriction, or premature birth. The safety profiles of different classes of AADs are discussed. Individualized treatment approaches and close monitoring of pregnant women prescribed AADs are essential to ensure optimal maternal and fetal outcomes.

## 1. Introduction

In the U.S., arrhythmias have been detected in 68 per 100,000 hospitalizations related to pregnancy. This value is likely to be underestimated, as some arrhythmic events may occur outside the hospital, or the arrhythmias are well-controlled because patients are already taking medications. Pregnancy-associated admissions with any arrhythmia exhibited greater overall frequencies of maternal or fetal complications and in-hospital mortality compared with all women population. The prevalence of tachyarrhythmias in pregnant women depends on age, being more frequent in women between 40 and 51 years, and cardiovascular comorbidities (such as hypertension, diabetes mellitus, obesity, and women who have experienced arrhythmias previously). African American women, particularly in the lowest income quartile, exhibit greater susceptibility to arrhythmias compared to Caucasian women [1]. 

Pregnancy is a significant stage in a woman’s life, marked by various structural transformations and hormonal shifts that can exert a considerable impact on the well-being of both the mother and her baby. Cardiovascular events and arrhythmias pose significant challenges to healthcare professionals during pregnancy [2].

The development of arrhythmias can be linked to physiological and hormonal changes endured by the cardiovascular system. These changes initially cause an increase in heart rate, cardiac output, ventricular end-diastolic volume, and atrial stretch, caused by an increase in intravascular volume. During pregnancy, there is a decrease in the parasympathetic autonomic system, while there is an increase in the activity of the sympathetic autonomic system at rest. This increased activity of the sympathetic nervous system can lead to the occurrence of abnormal automaticity, re-entry, or triggered activity [3].

Because antiarrhythmic drugs (AADs) will affect both the mother and the fetus, it is necessary to have complete knowledge of their safety, indications, and actions. Arrhythmias may provoke a sudden depletion of cardiac output with fatal consequences on the fetus [1] and the mother, with complications such as syncope, heart failure, cardiogenic shock, thromboembolism, and stroke. Using AADs to avoid anomalies in cardiac rhythm is fundamental to ensuring their overall health and well-being [4]. When using any AAD during pregnancy, one must consider the potential impact on the fetus. Some medications may cross the placenta or diffuse into breast milk, potentially reaching the fetus and generating adverse effects, such as teratogenic damage, growth restriction, or premature birth [5]. A tailored approach for every patient must be considered by choosing the correct AADs, timing, and dose, always considering mother and fetus safety [6]. For this reason, managing pregnancy arrhythmias requires collaboration among various healthcare professionals, including cardiologists, obstetricians, and maternal-fetal medicine specialists [7].

In conclusion, choosing the appropriate AADs in pregnancy is essential to guaranteeing maternal and fetal health.

## 2. Type of Arrhythmias

The various types of tachyarrhythmias in pregnancy have indeed different prevalence, therapies, and prognoses.

Atrial fibrillation (AF) or atrial flutter (AFL) are the most common arrhythmias in pregnancy, occurring in 31 per 100,000 pregnancies [8]. Due to the increasing prevalence of adult patients with congenital heart disease (CHD), caused by technological advances in diagnostic and surgical techniques and the higher propensity to supraventricular arrhythmias in this sub-population, in the foreseeable future, the incidence of AF/AFL during pregnancy in CHD patients is expected to increase [9]. This arrhythmia can result in rapid ventricular responses with severe hemodynamic consequences for both the mother and the fetus and is also associated with an increased risk of mortality (odds ratio (OR) 13.13, 95% CI 7.77–22.21; *p* < 0.0001) [7]. Additionally, pregnancy induces a hypercoagulable state, leading to an elevated risk of thromboembolism, particularly when associated with AF.

Supraventricular tachycardias (SVTs) are also quite frequent with a prevalence of 24 per 100,000 admissions. Atrioventricular nodal re-entry tachycardia (AVNRT) comprises 60% of SVTs, whereas atrioventricular re-entry tachycardias (AVRTs) make up around 10–20% of SVT occurrences [10]. Pregnancies among patients with SVT are associated with adverse obstetric and fetal outcomes. These include an elevated risk for severe maternal morbidity, cesarean delivery, low birth weight, preterm labor, fetal stress, and fetal abnormalities [11]. Nevertheless, pharmacological and ablative therapies are highly effective in treating these arrhythmias.

Sinus tachycardia is a benign arrhythmia and is considered a normal response to the physiological changes occurring during pregnancy and usually does not require treatment [10]. During pregnancy, there is a 25% increase in heart rate (HR). As a result, sinus tachycardia, especially during the third trimester, is quite common. However, doctors should pay attention to sinus tachycardia because it could be a symptom due to thyrotoxicosis, so in pregnancy, it is always useful to exclude hyperthyroidism to investigate other symptoms related to hyperthyroidism and perform imaging investigations that can help in the exclusion of this disease. 

Among the less common forms of SVT in pregnancy is ectopic atrial tachycardia, the prevalence of which, however, is much lower than AVNRT and AVRT [12].

Ventricular tachyarrhythmias (VTs) are extremely rare (16 per 100,000 admissions) so it is difficult to determine a rate estimation for ventricular arrhythmia. The most frequent VTs are:

- Premature ventricular complex (PVC), which is usually benign.
- Idiopathic VT, particularly arising from the right ventricular outflow tract.
- Inherited arrhythmia syndromes: Brugada syndrome and arrhythmogenic right ventricular cardiomyopathy (ARVC) are well-tolerated during pregnancy. Long QT syndrome shows a low risk of cardiac events during pregnancy, which is increased for 9 months postpartum compared to the healthy population [12]. For catecholaminergic polymorphic ventricular tachycardia (CPVT), beta-blockers and close monitoring are recommended.
- Drug-induced: certain medications, including some antiarrhythmic drugs, can cause QT interval prolongation and potentially lead to ventricular arrhythmias [13].
- Arrhythmia related to structural heart disease: Conditions such as ischemic heart disease, cardiomyopathy, congenital heart disease, myocarditis, or valvular heart disease can predispose pregnant women to ventricular arrhythmias.

Bradyarrhythmias are generally harmless for both mother and fetus and may cause symptoms in the presence of an underlying structural heart disease. Bradyarrhythmias are rare and typically do not pose a risk to the mother or fetus. First-degree atrioventricular blocks are generally harmless. Second-degree atrioventricular blocks are often asymptomatic and typically associated with structural heart disease or medication use. Complete atrial ventricular blocks (CAVBs) are uncommon and usually have favorable outcomes, especially in patients with congenital heart disease in which the CAVB is generally well tolerated [14]. In a few cases, a restricted group of patients may require temporary pacing and delay permanent pacemaker implantation until more than 8 weeks post-pregnancy. Establishing an association between bradycardia and symptoms is the key to determining whether a pacemaker is indicated [15].

## 3. Potential Risks Associated with the Use of AADs

The use of medications like AADs in pregnancy is correlated in the literature with an increased risk of adverse outcomes. Pregnant women who are prescribed antiarrhythmic medication, especially those with a greater risk of teratogenic effects, must be closely monitored throughout their entire pregnancy [15]. This periodic check may include regular electrocardiograms, targeted ultrasound examinations, prenatal checkups, targeted ultrasound examinations, and, if necessary, additional tests such as fetal echocardiography to assess fetal heart structure and function to rapidly identify any adverse effects (AEs) leading to immediate AAD discontinuation.

The use of AADs during the first trimester of pregnancy is often associated with a higher frequency of adverse outcomes because of organ development occurring in the initial 12 weeks. As a result, it is recommended to delay the use of AADs until the end of the first trimester [16]. The risk of teratogenic effects varies among different antiarrhythmic drugs. There have been many classifications regarding the safety of antiarrhythmic drugs to be used in pregnancy; even the FDA itself, which previously used a classification system in which a letter was assigned to each drug based on the demonstrated safety (from A representing no risk to X representing not recommended), drew up a new consensus in 2014 for the use of these drugs in pregnancy named the Pregnancy and Lactation Labeling Rule [15]. 

While the FDA classification provides a summary of evidence found in the scientific literature, the frequent use of some drugs in clinical practice has allowed for data to be obtained even in the absence of clinical studies. Therefore, despite certain drugs being categorized in the same FDA class, the scientific literature assigns them distinct safety classifications, as described in a recent review by Halpern et al. [17]. For instance, drugs categorized as FDA class C, including procainamide and adenosine, have comparable evidence levels yet display divergent safety profiles in clinical settings. Instead, Vaughan–Williams Class III antiarrhythmic agents like amiodarone (FDA class D) carry a heightened risk for congenital anomalies such as hypothyroidism, growth restriction, and neonatal bradycardia. It is worth noting that the risk of teratogenic effects generally diminishes with progressing weeks of pregnancy [6]. The use of antiarrhythmic drugs in pregnancy can also be associated with maternal side effects such as hypotension, bradycardia, or arrhythmia [3]. These side effects can hurt maternal health and may also require modifications to the medication regimen or additional monitoring.

## 4. Dealing with the Maternal-Fetal Cardiovascular System and Changes in Pharmacokinetics

Several changes occur in the female body that facilitate fetal growth but also substantially alter the physiological status of the woman. The maternal–fetal cardiovascular system is indeed a complex and interconnected network that ensures the proper exchange of nutrients, oxygen, and waste products between the mother and the developing fetus through the placenta [18].

The maternal and fetal cardiovascular systems are closely interconnected and must function in harmony to ensure the health and well-being of both the mother and the fetus. Any disturbances to this delicate equilibrium, for example, those triggered by maternal arrhythmias, have the potential to affect fetal development and result in complications.

During pregnancy, the increase in cardiac output and tidal volumes leads to hyperventilation and increased pulmonary blood flow. These changes enhance alveolar uptake and, therefore, should be considered when administering drugs by inhalation [19]. However, dose adjustments, for some drugs like anesthetics, are likely to be reduced during pregnancy [19].

The pharmacokinetics of pregnant patients are altered by the physical changes mentioned above. The increasing level of progesterone determines a delay in intestinal mobility in the small bowel that, along with nausea and vomits, could lead to a reduction in drug absorption [19]. However, some studies have not found differences in the bioavailability of drugs such as sotalol and propanolol compared to non-pregnant patients [20,21]. Maternal blood volume increases by approximately 40–50% to accommodate the needs of the growing fetus and placenta. This expansion is essential to maintaining adequate circulation and oxygen delivery to the fetus [22]. As the plasma volume of the total body rises, so does the lipophilic and hydrophilic drug’s volume of distribution; the drug’s initial concentration and peak concentration could decrease, thus demanding an increase in dosage [23]. To calculate the half-life of a drug in pregnancy, the variability of the volume of distribution, the binding to serum protein, the extraction rate, and the clearance must be taken into account. The hepatic extraction ratio (ER) consists of the fraction of a certain drug that is removed from the blood circulation by the liver; the ER is strongly dependent on the blood flow entity to the liver. Because the perfusion to the liver increases during pregnancy, drugs with a high ER such as propranolol and verapamil would be metabolized faster, requiring higher doses to obtain the same efficacy [24]. Moreover, while progesterone increases the activity of hepatic microsomal enzymes, the effects of estrogens on cholestasis may interfere with drug clearance; notably, they both competitively inhibit microsomal oxidase [24]. CYP3A and CYP2D6 are both induced in pregnancy: metoprolol levels, which are metabolized by CYP3A4, are lower in pregnant compared to nonpregnant women [25]. In a manner similar to the liver, the glomerular filtration rate also experiences an increase of 45% to 50% during pregnancy, owing to the increase in effective renal plasma flow from 50% to 85% [26]; so, drugs that are excreted primarily unchanged in the urine, such as penicillin, digoxin, and lithium, demonstrate enhanced elimination and lower steady-state serum concentrations. However, these changes are clinically insufficient and, therefore, require no alteration in the dose of the abovementioned drugs [19].

## 5. Classification of Antiarrhythmic Drugs (Vaughan Williams Classification)

Despite the new classification expressed by the FDA in the Pregnancy and Lactation Labeling Rule [15], we will adopt the Vaughan–Williams classification in this review since it is the most accredited and easiest to understand in the context of the topic discussed (Table 1). This classification divides antiarrhythmics into four classes: Na^+^ channel blockers (Class I), beta-adrenoceptor antagonists (Class II), drugs that predominantly block K^+^ channels and prolong the cardiac action potential duration (APD) without affecting intracardiac conduction (Class III), and non-dihydropyridine L-type Ca^2+^ channel blockers (Class IV). Later, Class I anti-arrhythmic drugs (AADs) were categorized into medications with intermediate (IA), fast (IB), and slow (IC) offsets of Na+ channel blockade kinetics.

Class I: Sodium channel blockers operate by blocking the fast sodium channels (Nav 1.5) expressed in atrial and ventricular cardiomyocytes, thereby reducing the rate of depolarization and the duration of the action potential. They also decrease conduction velocity, prolong the duration of action potential, and increase the effective refractory period. This class is subdivided into three subclasses based on their specific effects:

- Class Ia: These medications, including quinidine, procainamide, and disopyramide, moderately block sodium channels and have some impact on potassium channels, without affecting atrio-ventricular conduction. As a result, they are helpful for treating atrial and ventricular arrhythmias by slowing conduction velocity, prolonging the action potential duration, and increasing the effective refractory period. Quinidine and procainamide are considered second-line therapies for hemodynamically stable ventricular tachycardia. There is evidence supporting the effectiveness and safety of quinidine in preventing ventricular arrhythmias associated with Brugada syndrome due to periods of increased vagal tone [27].
- Class Ib: These drugs (e.g., lidocaine, mexiletine, and phenytoin) weakly block sodium channels and have minimal effects on action potential duration. They mainly prolong the post-repolarization refractoriness in ventricular cells, making them a suitable therapy for VT, particularly in the context of acute myocardial infarction or ischemia.
- Class Ic: These medications, including flecainide, propafenone, and moricizine, powerfully inhibit sodium channels while having little to no effect on the duration of action potentials. They decrease conduction velocity but only minimally affect the refractory period. These agents are employed in the treatment of atrial and ventricular arrhythmias, but caution should be exercised in patients with structural heart disease due to the risk of arrhythmia. Flecainide and similar drugs are recommended as initial treatment to terminate SVT in patients with known pre-excitation or a history of Wolf–Parkinson–White syndrome, as well as for long-term arrhythmia control.

Class II: Beta-blockers, such as propranolol, metoprolol, and atenolol, inhibit beta-adrenergic receptors in the heart, reducing sympathetic activity and leading to a decreased heart rate, slowed conduction velocity, and increased refractory period. These agents are effective in treating arrhythmias related to heightened sympathetic activity, including supraventricular tachycardia and atrial fibrillation. Beta-blockers are the preferred treatment for individuals experiencing recurring SVT without a known pre-excitation or Wolf–Parkinson–White history due to the potential danger of converting into pre-excited AF and subsequent conduction over the accessory pathway with high atrioventricular (AV) conduction. In the context of Long QT syndrome, propranolol and nadolol are the preferred medications [28].

Class III: Potassium channel blockers, such as Amiodarone, Sotalol, and Dofetilide, primarily block the potassium channels responsible for the repolarization phase of the action potential. Amiodarone also shows different effects on other cardiac channels, such as those of sodium and calcium. This extends the action potential duration and the effective refractory period, thereby making them effective for treating atrial and ventricular arrhythmias. 

Class IV: Calcium channel blockers, including verapamil and diltiazem, inhibit the L-type calcium channels situated in the heart. This results in decreased calcium influx that occurs during the plateau phase of the action potential leading to a slowed conduction velocity, particularly in the AV node. These agents are primarily used to treat supraventricular arrhythmias, such as atrial fibrillation and atrial flutter, by reducing the ventricular rate. These medications serve as the second course of treatment to chronically manage SVT in cases without known pre-excitation or Wolf–Parkinson–White Syndrome. Intravenous verapamil can cause significant hypotension and therefore is usually considered only when SVT is refractory to adenosine or beta-blockers, or when there are contraindications to these two preferred agents [15].

There are also drugs that were included in the Vaughan–Williams classification at a later stage and regrettably do not fit into the traditional categories. Therefore, they are labeled as unclassifiable antiarrhythmics.

Adenosine: this drug has a specific receptor on AV node cells, the “A1 receptors”, that inhibits adenylate cyclase by activating guanine nucleotide regulatory proteins G protein(s); these G proteins link the receptor to other cell membrane proteins, such as K channels. The activation of these receptors generates a K’ outward current (I_Kado)_ that provokes the hyperpolarization of AV nodal cells and a consequent AV block. This effect on the atrioventricular node is beneficial for all supraventricular tachycardia that involve this structure in their circuit, such as (AVNRT) and (AVRT)AVRT [29].

Digoxin: This is a cardiac glycoside that inhibits the cellular Na^+^/K^+^-ATPase. Its antiarrhythmic effects arise from its agonistic action on the parasympathetic nervous system, which slows electrical conduction in the atrioventricular node, thus reducing the heart rate. The blockage of Na^+^/K^+^-ATPase leads to increased calcium levels, which prolongs phase 4 and phase 0 of the cardiac action potential, thereby increasing the AV node’s refractory period [29].

### Safety Profiles of Antiarrhythmic Drugs in Pregnancy

Class Ia: Quinidine is the oldest class Ia drug used in pregnancy, with the first report of its use dating back to 1930. Adverse effects with quinidine use are rarely reported. Its use is considered safe during pregnancy [30]. Quinidine, procainamide, and disopyramide are documented to cross the placenta.

However, none of these drugs have been linked to teratogenic effects. The potential concerns of these drugs are torsade de pointes for quinidine, drug-induced lupus and pre-term labor for procainamide, and neonatal heart block for disopyramide. Notably, the serum level of procainamide must be periodically checked during its administration [31,32].

Class Ib: Lidocaine and Mexiletine can cross the placenta. The safety of lidocaine has been evaluated in a relevant number of studies and, taking into account the necessity to periodically check its serum levels, it does not affect the fetal heart rate, amniotic fluid pressure, or uteroplacental circulation within therapeutic doses [33,34]. Mexiletine is, instead, associated with lower Apgar scores and rare cases of fetal resorption; also, levels of Mexiletine must be periodically evaluated [35].

Class Ic: Neither Flecainide nor Propafenone is documented to cross the placenta. In the setting of flecainide use, this has usefulness in treating fetal arrhythmias without carrying any risk of intrauterine or neonatal toxicity [36]. Flecainide is one of the most-used antiarrhythmic drugs in pregnancy given its safety profile [37] and its effect on a wide variety of maternal arrhythmias, being effective both on SVTs and VTs [7].

On the other hand, there are limited data about Propafenone’s efficacy and safety. The pharmacokinetics of this substance involve a larger distribution of metabolites in the umbilical cord compared to plasma and the ability to reach breast milk [38]. It is considered not teratogenic, and its AEs are mainly found at 3–6 levels higher than the maximum recommended human dose [7].

Class II: The effects of this class of drugs during pregnancy are the most known. The more common adverse effects during this period are neonatal bradycardia (1.6% risk versus 0.5% of placebo) and neonatal hypoglycemia (4.3% risk versus 1.2% risk of placebo) [39]. Even if they can cross the placenta, they do not affect the risk of congenital malformation according to large registries [40]. Remarkably, propranolol is associated with a small risk to provoke fetal growth retardation [41]. Propranolol, metoprolol, nadolol, and pindolol are all safe in pregnancy. Atenolol, due to its decreased binding to proteins, is associated with more AEs, especially during fetal growth, and it must be avoided in pregnancy [42].

Class III: Amiodarone is known to cross the placenta [43]. The use of Amiodarone should be restricted to only those patients with arrhythmias that are resistant to other AADs and digoxin, as a last resort, due to its well-known negative effects such as fetal hypothyroidism, growth retardation, and prematurity [44].

Besides amiodarone, we have limited data on other III AADs: dronedarone, but also amiodarone, should be avoided in pregnancy or breastfeeding period because it is associated with vascular and limb abnormalities and cleft palate. The limited data regarding dofetilide discourage its use during pregnancy due to its inhibition of the rapidly delayed rectifier potassium channel leading to skeletal abnormalities and bradycardia [44,45]. The limited data regarding Ibutilide on the cardioversion of AF/FLA do not report AEs but are insufficient to assess its safety [45]. Animal studies reported a teratogenic risk at 4 times the recommended human doses [46]. Despite its capability to cross the placenta, sotalol is considered one of the safest drugs to use during pregnancy. The clearance and bioavailability of oral sotalol do not differ so much in pregnancy, even if its clearance after intravenous administration is more rapid in this status than in peripartum (6.6 h versus 9.3 h) [17]. Sotalol is not associated with any teratogenic issues and only has a slight risk of fetal hypoglycemia and bradycardia [45]. Sotalol and Amiodarone pose a risk of revealing ion channel disorders, such as QT syndrome, presenting the risk of a considerable QT interval prolongation and consequent increased risk of lethal torsade-de-pointes tachycardia. Regular electrocardiographic monitoring is required for patients undergoing sotalol or amiodarone therapy [27].

IV Class: the duration of antiarrhythmic activity is necessary while considering class IV drugs. If a short-acting antiarrhythmic is required, it is better to use adenosine. If a long-acting antiarrhythmic drug is required, verapamil should be chosen rather than diltiazem. This is because of the increased rate of preterm deliveries and decreased neonatal birth weight during chronic therapy with calcium blockers than placebo (23.8% versus 6.5%) [45]. Verapamil indeed has no significant risk of teratogenicity, but it can provoke fetal bradycardia and hypotension [7,8,9,10,11,12,13,14,15,16,17,18,19,20,21,22,23,24,25,26,27,28,29,30,31,32,33,34,35,36,37,38,39,40,41,42,43,44,45,46]. Meanwhile, animal studies indicate that diltiazem may cause fetal AEs [47].

Adenosine: This drug’s unique feature is its very short half-life of 10–30 s. For this reason, is a safe weapon to use in pregnancy, making it the first choice for terminating SVT after vagal maneuvers in a gestational setting [48,49]. Rare AEs include transient dyspnea, flashing, and chest pain. Notably, even if the adenosine deaminase action is reduced in pregnancy, the increase in intravascular volume manages to balance these effects.

Digoxin: Digoxin also represents one of the major cornerstones of antiarrhythmic therapy in pregnancy [50]. However, there is still concern about using it because, in pregnancy, many circulating digoxin-like fragments may compromise the accuracy of its serum level measurement; for this reason, both clinical signs of toxicity and the serum level of digoxin must be periodically checked [51]. Moreover, digoxin can also be used during lactation because the potential dose assumed by the newborn is not toxic [52].

## 6. Discussion

Two significant concerns remain when handling arrhythmias during pregnancy. The first is the lack of randomized clinical trials for AADs in pregnancy. All the information we possess regarding AADs has been collected from case series or even case reports. Hence, it is reasonable to start any AAD at the lowest effective dose in the presence of an electrophysiologist trained in managing pregnancy-related arrhythmias. Following this, the dosage can be gradually increased while closely monitoring the safety and effectiveness of the drug in each patient. The second aspect of concern is represented by the change in patients who go through pregnancy nowadays [53]. Women are now conceiving at older ages, with higher prevalences of coexisting congenital heart disease and cardiovascular risks that would have precluded pregnancy in previous eras but that may now be manageable. Taking this into account, cardiovascular mortality is the first cause of death in the pregnancy period [54] with an increase in its incidence in the last two decades [55,56].

Considering the complexity of the arrhythmic burden and the possible harmful effects of arrhythmias on both the mother and the fetus, a multidisciplinary approach is required. Cardiologists, pediatricians, and neonatologists must collaborate to find the “golden pill” for the specific situation and always consider direct electrical cardioversion and catheter ablation in the management of arrhythmias.

## 7. Conclusions

The use of AADs in pregnancy still represents a challenge. The changing profile of the typical expecting mother, the lack of randomized clinical trials, and the constant rise of cardiovascular comorbidities during pregnancy are the main aspects to consider. Currently, adenosine, flecainide, sotalol, and digoxin are the four safest and most effective drugs we can use to treat pregnancy-related arrhythmias.

## Figures and Tables

**Table 1:** The Classification of Antiarrhythmic Drugs.

Safety	Vaughan Williams Class	Drugs	FDA Category	Placental Transfer	Fetal Risk	Use in Fetal Treatment
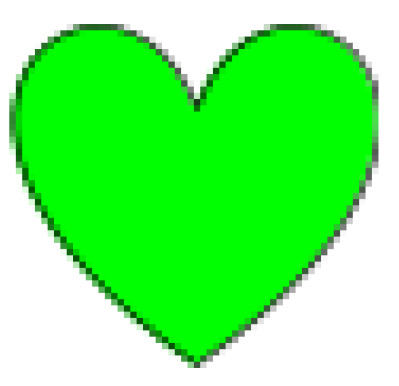	-	Adenosine	C	Unknown	Minor	No
	IB	Lidocaine	B	Yes	Minor	No
	-	Digoxin	C	Yes	Minor	Yes
	II	Metoprolol/Propranolol	C	Yes	Minor	No
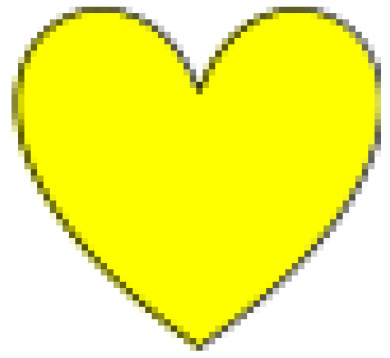	IA	Procainamide	C	Yes	Minor	No
	IA	Quinidine	C	Yes	Minor	No
	IB	Mexiletine	C	Yes	Minor	No
	IC	Flecainide	C	Yes	Minor	Yes
	IC	Propafenone	C	Yes	Minor	No
	III	Sotalol	B	Yes	Minor	Yes
	IV	Verapamil	C	Yes	Moderate	No
	IV	Diltiazem	C	No	Moderate	No
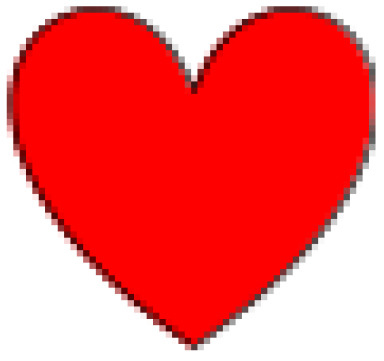	II	Atenolol	D	Yes	Major	No
	III	Amiodarone	D	Yes	Major	No

Antiarrhythmic drugs divided using the Vaughan-Williams classification and the FDA (Food and Drug Administration) classification, including information on crossing the placenta, fetal risk, and potential use to treat the fetus. The hearts are color-coded based on their possible safety as determined by clinical practice: green for considered safe; yellow for uncertain safety due to lack of data; red for use contraindicated.

## Data Availability

No new data were created or analyzed in this study.
